# Current management and novel agents for malignant melanoma

**DOI:** 10.1186/1756-8722-5-3

**Published:** 2012-02-14

**Authors:** Byung Lee, Nikhil Mukhi, Delong Liu

**Affiliations:** 1 Division of Hematology and Oncology, New York Medical College and Westchester Medical Center, Valhalla, New York 10595, USA

## Abstract

Advanced malignant melanoma remains a challenging cancer. Over the past year, there have been 3 agents approved for treatment of melanoma by Food and Drug Administration. These include pegylated interferon alpha-2b for stage III melanoma, vemurafenib for unresectable or metastatic melanoma with BRAF V600E mutation, and ipilimumab for treatment of unresectable or metastatic melanoma. This review will also update on the development of novel agents, including tyrosine kinase inhibitors and adoptive cellular therapy.

## Introduction

Melanoma is a process when normal melanocytes undergo a malignant transformation [1]. Over the past year, there have been 3 agents approved for treatment of melanoma by Food and Drug Administration [2-4]. These include pegylated interferon alpha-2b for stage III melanoma [2], vemurafenib for unresectable or metastatic melanoma with BRAF V600E mutation [3], and ipilimumab for treatment of unresectable or metastatic melanoma [4]. The American Cancer Society estimates that 68,130 new melanomas was diagnosed and approximately 8,700 people died from melanoma in 2010 [5]. The incidence of Melanoma has increased to 22.52 per 100,000 in 2008 from 7.89 per 100,000 in 1975 [6]. Clinical and epidemiological data suggests increased incidence of melanoma in people with extensive or repeated exposure to sunlight [7]. Individuals with family history of melanoma are at significantly higher risk for developing this malignancy, representing 5-12% of all reported cases [8]. The risk of melanoma is associated with high nevi count [9]. One clinically dysplastic nevus is associated with 2 fold risk and 10 or more have a 12 fold increased risk of developing malignant melanoma [9]. Biopsy of a suspicious lesion is necessary for an accurate diagnosis and for optimal staging.

## Management

### Management of Clinically Localized Melanoma

Wide local excision is the treatment of choice for primary melanoma [10]. The proper resection margin is based on the thickness of the lesion. According to NCCN guidelines, melanoma with 1.0 mm or less (T1), wide excision with a 1.0 cm margin is recommended. For localized melanomas between 2 and 4 mm thick (T3), a 2 cm excision is suggested [10]. For thicker melanomas > 4 mm(T4), The U.S. Intergroup Melanoma Surgical Trial established that a 2-cm margin is adequate. Thick melanomas are associated with a higher risk of nodal and distant metastases. However, more extensive resection is unlikely to substantially change the outcome [1].

### Sentinel Lymph node biopsy

Multicenter Selective Lymphadenectomy Trial evaluated the usefulness of sentinel-node biopsy (SLNB) in the identification of patients with clinically occult nodal metastases and to examine the clinical effect of immediate, complete lymphadenectomy in patients with tumor-positive sentinel lymph nodes. Among 1269 patients with intermediate thickness primary melanoma, the mean estimated 5 year disease free survival was significantly higher in the node biopsy group compared to the observation group at 5 years (78.3% vs. 73.1%; P = 0.009)[11]. Among patients with nodal metastasis, the 5 year survival rate was higher among those who had immediate lymphadenectomy performed than among those in whom lymphadenectomy was delayed (72.3% vs. 52.4%; P = 0.004). Five year melanoma survival rates were similar between two groups (87.1% vs. 86.6%)[11]. SLNB is currently recommended for melanomas > 1.0 mm thick or greater, 1.0 mm or less with ulceration or mitotic rate more than or equal to 1 per mm2 and resectable solitary in-transit stage III disease.

### Adjuvant Systemic Therapy

#### High Dose Interferon

It is well known that the immune system responds naturally to melanoma and that immune modulation can be therapeutic for advanced melanoma [1]. The effect of interferon alfa (IFNα) as a single agent or in combination has been explored in various clinical trials. A randomized control study by Kirkwood et al of IFN alpha-2b administered at 20 MU/m2/d intravenously for 1 month and 10 MU/m2 three times per week subcutaneously for 48 weeks was compared to observation alone, conducted by the Eastern Cooperative Oncology Group (ECOG) 1684 in 287 patients who had > 4 mm thick melanoma or were node positive (stage IIb/IIc/III)[12]. A remarkable prolongation of disease free survival (DFS) (from 1.0 to 1.7 years P = .0023, one-sided) and prolongation of overall survival (OS) (from 2.8 to 3.8 years P = .0237, one-sided) was noticed with IFN alpha-2b therapy in this trial. The increase in median DFS and OS that results from this therapy is correlated with a 42% improvement in the fraction of patients who continues to be disease-free after treatment with IFN (from 26% to 37%) in comparison to observation [12]. On the basis of the results of the ECOG 1684 trial, the use of high-dose IFN2b for the adjuvant therapy of patients with stage IIB-III melanoma was approved by FDA in 1995 [1].

ECOG 1690 was a prospective, randomized, three-arm intergroup trial which assessed the efficacy of high-dose IFN (HDI) alpha-2b (20 MU/m2 IV for 5 d/wk for 4 weeks; 10 MU/m2 SC 3 times/wk for 48 weeks) for 1 year and low-dose IFN (LDI) alpha-2b (3 MU SC 3 times/wk for 2 years) for 2 years versus observations (Obs) in high-risk (stage IIB and III) melanoma. The estimated 5 year RFS rates for the HDI, LDI, and Obs arms were 44%, 40%, and 35%, respectively [13]. Hence RFS benefit of IFN alpha2b is dose-dependent and significant for HDI. However, unlike ECOG 1684, the ECOG 1690 failed to show any difference in OS between different arms [13].

Additional pooled analysis of E1684, E1694, and E18952 confirmed an improvement in RFS in patients with high risk resected melanoma (P = .006) but failed to demonstrate a significant improvement in overall survival. There are ongoing studies regarding modified dosing regimens, defining mechanism of action, and finding more effective combination regimens incorporating vaccines.

#### Pegylated Interferon

European Organization for Research and Treatment of Cancer (EORTC) 18991 organized a study on stage III melanoma patients, evaluating the efficacy and toxicity of long term pegylated interferon (PEG-IFN) vs. Observation. PEG-IFN (Induction at 6 μg/Kg/wk, sc, 8 weeks; followed by maintenance at 3 μg/Kg/wk, sc) therapy for 5 years was compared to Obs. in 1256 patients with stage III melanoma (any T, N1-2, M0 without in-transit metastases). Four-year relapse-free survival was significantly better in the interferon group in comparison to the observation group (45.6% vs. 38.9%). However, there was no significant effect of pegylated interferon on OS [2].

Based on this data from EORTC 18991, pegylated interferon received approval by the FDA in 2011 as adjuvant therapy for melanoma with nodal involvement, demonstrating a sustained impact on RFS in patients with lymph node positive melanoma. Weekly dose of pegylated interferon appears more convenient than 3 times weekly injection of high dose interferon [2].

When treating patients with adjuvant high-dose interferon or pegylated interferon, it should be based on individualization of each patient factoring in past medical history, performance status, and co-morbidities. According to NCCN guidelines, adjuvant treatment options for stage IIB or IIC melanoma (2.0-4.0 mm with ulceration or > 4.0 mm) include clinical trials, observation, or high-dose interferon. For stage III disease (positive nodes), options include clinical trials, observation or interferon alfa. In addition to interferon alfa, pegylated interferon is an option in completely resected stage III melanoma. Currently, high-dose interferon and pegylated interferon is category 2B due to its limited benefits compared to risks of possible side effects.

#### Adjuvant Radiation Treatment

Early stage melanoma is often cured with primary surgery. However, there are situations in which adjuvant radiation therapy (RT) should be considered. According to NCCN guidelines, the following situations should be considered for adjuvant RT: those with inadequate margins, high risk nodal disease such as multiple positive nodes, larger nodes, those with macrovascular extranodular soft tissue extension, melanoma involving cervical lymph nodes, and desmoplastic neurotropic melanoma which tends to be more aggressive.

#### Treatment of Unresectable Metastatic (Stage IV) Melanoma

The incidence of metastatic melanoma has increased over the past three decades, and the death rate continues to rise faster than the rate with most cancers [14]. The survival rate for patients with metastatic melanoma is low, with an expected 2-year survival rate of 10 to 20%. In the United States alone, an estimated 8700 persons died from melanoma in 2010 [5].

#### Surgery

A small group of patients may potentially benefit from surgery for distant metastatic (stage IV) melanoma. The benefit can be palliative in some patients and may be potentially curative in selected few cases [1]. Situations in which the benefit of surgery is definitive include: anemia secondary to occult bleeding from intestinal metastasis, small bowel metastasis resulting in bowel obstruction, cutaneous or subcutaneous ulceration due to metastasis, neurologic symptoms as a result of lymph node metastasis, symptomatic brain metastasis, and life-threatening hemorrhage due to metastasis. There is no standard adjuvant therapy post resection of metastatic melanoma [1].

#### Chemotherapy

In 1975, dacarbazine (DTIC) became the first chemotherapeutic agent approved by FDA for the treatment of metastatic melanoma. The response rates with dacarbazine were 15-25%, with median response durations of 5-6 months, but less than 5% of complete responses [15]. Temozolomide and fotemustine have also been compared with DTIC [16,17]. A number of regimens combining dacarbazine with other cytotoxic agents, tamoxifen, or interferon alfa have shown promising response rates in single-institution phase 2 trials and potential survival advantages in phase 3 trials. However, despite extensive investigation, no randomized controlled trials have proven these approaches to be superior to dacarbazine alone [15].

### Immunotherapy for Systemic Treatment of Stage IV Melanoma

Immunotherapy for melanoma and other cancer types have been widely studied [18]. Interleukin-2, monoclonal antibodies, and cellular therapy have been in active clinical and preclinical trials.

#### High-dose Interleukin-2

The intravenous administration of high-dose interleukin-2 (IL-2) constitutes an effective treatment for patients with metastatic melanoma and the treatment capable of providing long-term complete responses and potential cure in these patients [1]. IL-2 alone, is ineffective on cancer cells directly, and its antitumor activity is dependent on its ability to modulate immunologic responses in the host [19]. In the initial study of high-dose IL-2 by Atkins et al, the overall response rate was 16% (95% CI, 12% to 21%), median duration of response for all responders was 8.9 months, fifty-eight percent of the responders remained progression-free at 12 months, the median survival duration for all patients in the study was 11.4 months, and there were no relapses in responding patients after 30 months [19]. The uniqueness of IL-2 therapy is its capability to mediate durable complete responses in patients with widespread metastatic disease. With a median follow-up of 62 months, 47% of the responding patients were still alive [19]. Although chemotherapy regimens and high-dose IL-2 show similar regression rates, only high-dose IL-2 is capable of achieving the level of durable complete responses [1]. High-dose IL-2 was approved by the FDA for the treatment of metastatic melanoma in January 1998 due to its ability to mediate durable responses. Most of the severe toxicities resembled the clinical manifestations of septic shock such as hypotension, supraventricular tachycardia, and respiratory distress syndrome. Nausea, vomiting, and diarrhea were common, but life-threatening gastrointestinal side effects were rare. Mental status changes were also common and could be severe. Although elevations of creatinine levels were common, all patients were able to recover renal function after completion of therapy. Infections were reported in 15% of patients, with life-threatening infections or sepsis occurring in 3%[19].

### Novel Agents for Targeted Therapy

#### Ipilimumab

**Figure 1:**
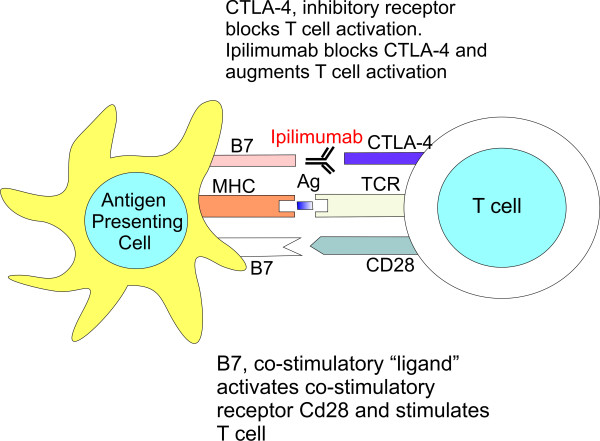
**The mechanism of action of anti-CTLA-4 monoclonal antibody**.

Cytotoxic T lymphocyte-associated antigen 4 (CTLA-4) is an immunomodulatory molecule, expressed on both CD8+ and CD4+ T cells, allowing peripheral tolerance by suppressing T-cell activation and proliferation (Figure 1)[1]. Activation through CTLA-4 results in a reduction in T-cell responsiveness and increases the threshold for T-cell activation [1]. Blockade of CTLA-4 function results in enhancement of antitumor immunity, since tumors primarily express non-mutated, self-antigens. Closer look on these findings in clinical trials revealed that the utilization of an anti-CTLA-4 blocking antibody mediated objective responses in approximately 15% of patients with metastatic melanoma [20].

Ipilimumab, a fully humanized IgG1 monoclonal antibody, blocks CTLA-4, thus amplifying T-cell activation and proliferation [21]. Ipilimumab has shown activity in patients with metastatic melanoma when it was used as monotherapy in phase 2 study [22].

A dose-dependent response was seen in group receiving ipilimumab at a dose of 10 mg per kilogram in a phase 2 study [22]. In the randomized phase 2 study by Hersh et al combination of DTIC and Ipilimumab was associated with durable objective responses without new adverse events [23].

Ipilimumab has also demonstrated activity when combined with other agents, including cancer vaccines [20]. One well-studied cancer vaccine consists of Human Leukocyte Antigen (HLA) A-201 restricted peptides derived from the melanoma protein, glycoprotein 100 (gp100). Monotherapy with gp100 generates immune responses but has limited antitumor activity [24]. With no proven standard of care, Hodi et al. used gp100 as an active control for phase 3 study, which evaluated whether ipilimumab with or without gp100 improves overall survival, as compared with gp100 alone, among patients with metastatic melanoma [4]. The median overall survival in the ipilimumab-plus-gp100 group was 10.0 months vs. 10.1 months in the ipilimumab-alone group and median overall survival is 6.4 months in the gp100-alone. OS rates in the ipilimumab-plus-gp100 group, the ipilimumab-alone group, and the gp100-alone group, respectively, were 43.6%, 45.6%, and 25.3% at 12 months, 30.0%, 33.2%, and 16.3% at 18 months, and 21.6%, 23.5%, and 13.7% at 24 months [4]. According to Hodi et al., the effect of ipilimumab on overall survival was independent of age, sex, baseline serum lactate dehydrogenase levels, metastasis stage of disease, and previous exposure interleukin-2 therapy [4].

Dacarbazine has been the drug most frequently compared with new agents or combination therapies in randomized trials involving patients with melanoma, although it has never been shown to improve survival in randomized, controlled studies [25]. Phase 3 study was conducted by Wolchok et al. to determine whether ipilimumab (at a dose of 10 mg per kilogram) plus dacarbazine, as compared with dacarbazine and placebo improves overall survival in patients with previously untreated metastatic melanoma [26]. The median overall survival in the ipilimumab-dacarbazine group was 11.2 month and 9.1 months in the dacarbazine group, with estimated survival rates in the two groups, respectively, of 47.3% and 36.3% at 1 year, 28.5% and 17.9% at 2 years, and 20.8% and 12.2% at 3 years. There was a 24% decrease in the risk of progression in the ipilimumab-dacarbazine group as compared with the dacarbazine group (hazard ratio for progression, 0.76; P = 0.006)[26]. However, there was a higher incidence in the ipilimumab-dacarbazine group than in the dacarbazine group included elevation of alanine aminotransferase levels (in 33.2% of patients vs. 5.6%), elevation of aspartate aminotransferase levels (29.1% vs. 5.6%), diarrhea (36.4% vs. 24.7%), pruritus (29.6% vs. 8.8%), and rash (24.7% vs. 6.8%)[26]. Grade 3 or 4 adverse events occurred in 56.3% of patients receiving ipilimumab plus dacarbazine and in comparison 27.5% of patients receiving placebo plus dacarbazine (P < 0.001). The most common grade 3 or 4 immune-mediated adverse reaction was immune-mediated hepatitis, which was observed in 78 patients in the ipilimumab-dacarbazine group (31.6%) and in 6 patients in the dacarbazine group (2.4%). Grade 3 or 4 immune-mediated enterocolitis was detected in 12 patients in the ipilimumab-dacarbazine group (4.9%) and no patients in the dacarbazine group [26]. The hepatic complications were generally reversible. Patients who received glucocorticoids or other immunosuppressant agents after the emergence of high-grade immune-mediated hepatitis were 80.8% in the ipilimumab-dacarbazine group and 33.3% in the dacarbazine group. No patient died from the complications of immune-mediated hepatitis or enterocolitis during the course of the study [26]. Based on phase 3 studies by Hodi et al, and Wolchok et al, ipilimumab was approved by FDA in 2011 for treatment of unresectable or metastatic melanoma at a dose of 10 mg per kilogram every 3 weeks for 4 doses.

### Adoptive T-Cell Therapy

Adoptive T cell therapy (ATCT) consists of isolating tumor reactive lymphocytes from a cancer patient, growing and activating them in vitro, and infusing them back into same patient. ATCT was first described in 1988. Rosenberg et al used autologous tumor-infiltrating lymphocytes (TIL) derived from freshly resected metastatic melanomas.

Morgan et al did the first clinical trial that demonstrated anti-tumor responses by adoptive cell transfer of lymphocytes transduced with retroviral encoding T-cell receptors (TCR). The first clinical trial to successfully mediate the regression of human cancer by ATCT used genetically engineered autologous lymphocytes [27]. Sixteen patients were treated in the trial. These patients had lymphocytes with a TCR reactive with the melanoma-associated antigen recognized by T cells (MART1), which was isolated from highly reactive TIL. Two patients underwent regression of liver and lung hilum metastases respectively and both remained disease free 2 years later. TCRs with far greater affinity for the MART1 melanoma antigen have been identified and are now being evaluated in clinical gene therapy trials [28].

Phage display techniques have been used to generate TCRs with 106 times the affinity of a natural TCR directed against the cancer- testis antigen NY-ESO-1 (present on common epithelial cancers and 10-50% of melanomas). Robbins et al recently conducted a clinical trial targeting the NY-ESO-1 antigen with TILs. The study showed objective responses in 6 out of 11 patients, of which 2 patients had complete response [28]. These studies suggest ATCT can be a potent and effective therapy for malignant melanoma. A major problem is that it is highly personalized and labor-intensive which makes its commercial use difficult.

### Vemurafenib

**Figure 2:**
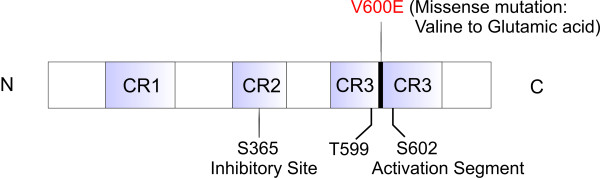
**Molecular structure of B-RAF and the V600E (valine to glutamic acid) missense mutation**. C: carboxyl-terminal; N: amino-terminal.

A search for mutations in the mitogen-activated protein (MAP) kinase pathway in various common cancers by Davies et al, revealed that 40% to 60% of melanomas, and a 7 to 8% of all cancers carry an activating mutation in the gene encoding the serine-threonine protein kinase BRAF (Figure 2)[29,30]. Although other activating mutations are known (e.g., BRAF V600K and BRAF V600R), approximately 90% of these mutations result in the substitution of glutamic acid for valine at codon 600 (BRAF V600E)[5]. This BRAF mutation makes it constitutively activated and leads to constitutive activation of the MAP kinase pathway [31].

Vemurafenib is a potent and specific inhibitor of BRAF with the V600E mutation. It has marked antitumor effects against melanoma cell lines with the BRAF V600E mutation only. It is inactive in the cell lines with wild type BRAF [32].

Flaherty et al. conducted a Phase I and II trials for vemurafenib study in patients with unresectable, previously untreated stage IIIC or stage IV melanoma that tested positive for the BRAF V600E mutation [31]. A phase 1 trial established the maximum tolerated dose to be 960 mg twice daily which showed responses against the tumor [31]. A phase 2 trial involving patients who had received previous treatment for melanoma with the BRAF V600E mutation displayed a confirmed response rate of 53%, with a median duration of response of 6.7 months. The levels of phosphorylated extracellular signal-regulated kinase (ERK), cyclin D1, and Ki-67 were markedly reduced at day 15 as compared with baseline in all specimens examined. This study proposed that vemurafenib inhibited the MAP kinase pathway, resulting in decreased cyclin D1 levels and decreased proliferation. A marked decrease in tumor uptake of FDG was noted at day 15 in virtually all patients [31].

Subsequently, Phase III trial was conducted in 680 patients with previously untreated, unresectable stage IIIC or stage IV melanoma with BRAF V600E mutations. The patients were randomized to vemurafenib (960 mg po bid) or dacarbazine (1,000 mg/m^2^, IV, q3w)[33]. Patients were evaluated for tumor responses after weeks 6, 12, and then q9 weeks [34]. There was an increase in median survival from 8 months for dacarbazine to 12.3 months for vemurafenib [3]. Crossover of patients in the dacarbazine group was allowed subsequently to vemurafenib group, and the protocol was amended accordingly. Median follow-up for the interim analysis was 3.8 months for patients in the vemurafenib group and 2.3 months for those in the dacarbazine group [3]. A total of 672 patients were evaluated for OS. At 6 months, OS was 84% in the vemurafenib group compared to 64% in the dacarbazine group [3]. Estimated median progression-free survival (PFS) in the vemurafenib group and in the dacarbazine group was 5.3 months and 1.6 months respectively [3].

The most common adverse events in the vemurafenib group were cutaneous events, arthralgias, and fatigue; photosensitivity skin reactions of grade 2 or 3 were seen in 12% of the patients. Among patients treated with vemurafenib, 18% were reported to have at least one squamous-cell carcinoma of the skin or keratoacanthoma [3]. Median time to the appearance of a cutaneous squamous-cell carcinoma was 8 weeks; most of the carcinomas were resected, and in no case did they lead to treatment discontinuation. These squamous-cell carcinoma and keratoacanthoma type are well-differentiated tumors with very low invasive potential and virtually no metastatic capabilities [31]. The mechanism of the increase in cutaneous neoplasia associated with vemurafenib administration is unclear, but it is hypothesized to involve the activating effect of vemurafenib on pre-neoplastic cells in which wild-type BRAF is further primed by upstream pathway activation [3].

Vemurafenib displayed a relative reduction of 63% in the risk of death and of 74% in the risk of tumor progression in untreated, unresectable stage IIIC or stage IV melanoma with the BRAF V600E mutation, in comparison with treatment with dacarbazine [3]. Vemurafenib 960 mg, orally administered twice daily was approved by FDA in 2011 to treat patients with metastatic or unresectable melanoma.

### KIT Mutation

Melanomas present on mucosal membranes, acral skin (soles, palms, and nail bed), and on chronic sun-induced damage areas often do not have mutations within the genes of MAP kinase pathway such as BRAF and NRAS, which are commonly mutated in intermittent sun-exposed areas. This raises the question of other aberrations in MAP kinase system. Curtin et al studied 102 patients with primary melanoma for DNA copy number aberrations specific to melanoma subtypes where mutations in BRAF and NRAS are infrequent. He found a narrow amplification on 4q12 and analyzed candidate genes within. Mutations and/or copy number of KIT were increased in 39% of mucosal, 36% of acral, and 28% of melanomas on chronically sun-damaged skin, but 0% on melanomas of non-chronic sun exposed skin [35].

KIT mutations are frequently found in gastrointestinal stromal tumors (GIST) and have been shown to be highly sensitive to imatinib, a tyrosine kinase inhibitor (TKI) targeting ABL, PDGF-R, and KIT, thus providing a potential for targeted therapy in patients with KIT mutations in melanoma. Two new TKI agents have also been approved [36]. The initial Phase II trials using imatinib showed no objective benefit and poor survival rate in patients with metastatic melanoma [37].

More recently Carvajal et al conducted an open-label, phase 2 trial, with advanced unresectable melanoma with KIT alterations and amplifications. From this trial, 28 patients were administered with imatinib at a dose of 400 mg, twice daily. Two complete responses lasting 94 weeks and ongoing, 2 durable partial responses lasting 53 and 89 (ongoing) weeks, and 2 transient partial responses lasting 12 and 18 weeks among the 25 evaluable patients were observed. The overall durable response rate was 16%, with a median time to progression of 12 weeks, and a median overall survival of 46.3 weeks [38]. There is a similar phase II study conducted in China by Guo et al. In this phase II trial, 43 patients with metastatic melanoma harboring c-KIT mutations were treated with imatinib at 400 mg daily until disease progression or intolerable toxicities with a median follow up of 12 months. It showed 10 patients (23.3%) displayed partial response and 13 patients (30.2%) had stable disease with 18 of 43 patients (41.9%) showing regression of tumor mass. Based on these data, imatinib has shown promising results as a therapeutic agent in metastatic melanoma patients with c-KIT mutations [30].

## Conclusion and future directions

Pegylated interferon alpha-2b, vemurafenib and ipilimumab were approved for treatment of melanoma by Food and Drug Administration in the past year. Novel agents, including tyrosine kinase inhibitors and adoptive cellular therapy, are being explored. It is foreseeable that agents with novel mechanisms of action will be studied for this challenging malignancy [39-41].

## Abbreviations

BRAF: serine/threonine protein kinase; SLNB: sentinel lymph node biopsy; IFNα: Interferon alfa; ECOG: eastern cooperative oncology group; DFS: disease free survival; OS: overall survival; FDA: food and drug administration; HDI: high dose interferon; MU: million units; LDI: low dose interferon; Obs: observations; RFS: relapse free survival; EORTC: European organization for research and treatment of cancer; PEG-IFN: pegylated interferon; DTIC: dacarbazine: IL-2: interleukin 2; CTLA: cytotoxic T lymphocyte antigen; HLA: human leukocyte antigen; ATCT: adoptive T cell therapy; TIL: tumor infiltrating lymphocytes; TCR: T cell receptors; MART: melanoma associated antigen recognized by T cells; MAP: mitogen activated protein; ERK: extracellular signal regulated kinase; GIST: gastrointestinal stromal tumor; TKI: tyrosine kinase inhibitor; PDGF: platelet derived growth factor.

## Competing interests

The authors declare that they have no competing interests.

## Authors' contributions

All authors participated in concept design, data collection and analysis, drafting and critically revising the manuscript. All authors read and approved the final manuscript.
